# The International Psychosis Epidemiology Consortium Virtual Databank—A Platform for Data Harmonization and Federated Analysis of Psychosis Cohorts

**DOI:** 10.1093/schbul/sbaf094

**Published:** 2025-07-17

**Authors:** Vera Brink, Natalia Tiles-Sar, Hannah Jongsma, Catheleine van Driel, Craig Morgan, Bart Lestestuiver, Ellen Visser, Erwin Veermans, Morris A Swertz, Erna van ’t Hag, Carsten Hjorthøj, Merete Nordentoft, Nikolai Albert, Stynke Castelein, Wim Veling

**Affiliations:** University of Groningen, University Medical Center Groningen, University Center Psychiatry (CC 11), Hanzeplein 1, PO Box 30.001, 9700 RB Groningen, The Netherlands; University of Groningen, University Medical Center Groningen, University Center Psychiatry (CC 11), Hanzeplein 1, PO Box 30.001, 9700 RB Groningen, The Netherlands; University of Groningen, University Medical Center Groningen, University Center Psychiatry (CC 11), Hanzeplein 1, PO Box 30.001, 9700 RB Groningen, The Netherlands; Veldzicht Centre for Transcultural Psychiatry, Ommerweg 67, PO Box 20, 7707 ZG Balkbrug, The Netherlands; University of Groningen, University Medical Center Groningen, University Center Psychiatry (CC 11), Hanzeplein 1, PO Box 30.001, 9700 RB Groningen, The Netherlands; ESRC Centre for Society and Mental Health, King’s College London, London, United Kingdom; Department of Health Service and Population Research, Institute of Psychiatry, Psychology, and Neuroscience, King’s College London, 18 De Crespigny Park, SE5 8AF London, United Kingdom; University of Groningen, University Medical Center Groningen, University Center Psychiatry (CC 11), Hanzeplein 1, PO Box 30.001, 9700 RB Groningen, The Netherlands; University of Groningen, University Medical Center Groningen, University Center Psychiatry (CC 11), Hanzeplein 1, PO Box 30.001, 9700 RB Groningen, The Netherlands; University of Groningen, University Medical Center Groningen, Rob Giel Research Center, PO Box 30.001, 9700 RB Groningen, The Netherlands; University of Groningen, University Medical Center Groningen, University Center Psychiatry (CC 11), Hanzeplein 1, PO Box 30.001, 9700 RB Groningen, The Netherlands; University of Groningen, University Medical Center Groningen, Rob Giel Research Center, PO Box 30.001, 9700 RB Groningen, The Netherlands; University of Groningen, University Medical Center Groningen, Department of Genetics, PO Box 30.001, 9700 RB Groningen, The Netherlands; University of Groningen and University Medical Center Groningen, Genomics Coordination Center, PO Box 30.001, 9700 RB Groningen, The Netherlands; University of Groningen, University Medical Center Groningen, University Center Psychiatry (CC 11), Hanzeplein 1, PO Box 30.001, 9700 RB Groningen, The Netherlands; Copenhagen Research Center for Mental Health—CORE, Mental Health Center Copenhagen, Copenhagen University Hospital, Gentofte Hospitalsvej 15, 4, DK-2900 Hellerup, Denmark; University of Copenhagen, Department of Public Health, Section of Epidemiology, Øster Farimagsgade 5, DK-1353 Copenhagen K, Denmark; Copenhagen Research Center for Mental Health—CORE, Mental Health Center Copenhagen, Copenhagen University Hospital, Gentofte Hospitalsvej 15, 4, DK-2900 Hellerup, Denmark; Copenhagen Research Center for Mental Health—CORE, Mental Health Center Copenhagen, Copenhagen University Hospital, Gentofte Hospitalsvej 15, 4, DK-2900 Hellerup, Denmark; OPUS, Mental Health Center Copenhagen, University Hospital Copenhagen, Lersø Parkalle 112, Copenhagen East, Denmark; University of Groningen, University Medical Center Groningen, Rob Giel Research Center, PO Box 30.001, 9700 RB Groningen, The Netherlands; Lentis Psychiatric Institute, Lentis Research, Hereweg 80, 9725 AG Groningen, The Netherlands; University of Groningen, Faculty of Behavioral and Social Sciences, Department of Clinical Psychology and Experimental Psychopathology, Grote Kruisstraat 2/1, 9712 TS Groningen, The Netherlands; University of Groningen, University Medical Center Groningen, University Center Psychiatry (CC 11), Hanzeplein 1, PO Box 30.001, 9700 RB Groningen, The Netherlands

**Keywords:** catalog, FAIR, schizophrenia, remote, federated, cohort

## Abstract

**Background:**

Harmonization of research methodology, measures, and existing cohort data is needed to advance the field of psychosis epidemiology. The International Psychosis Epidemiology Consortium (IPEC) has been initiated to create a data-sharing platform for psychosis cohorts globally and provide infrastructure for data harmonization. This profile paper describes the design and data harmonization process, and the technical, ethical, and legal steps taken to set up the IPEC virtual databank, as well as the organizational structure we developed for IPEC.

**Study Design:**

An international group of researchers, collaborating in the Schizophrenia International Research Society—Epidemiology Research Harmonization Group, drafted inclusion and exclusion criteria for participating cohorts and selected, among others, sociodemographic, socioeconomic, and clinical variables for harmonization. Drawing upon current guidelines for data harmonization, a guideline specifically for psychosis cohorts and a software architecture for federated analysis were developed. Finally, as proof of principle, all steps of data harmonization were applied to 2 cohorts, and summary statistics on core variables were calculated.

**Study Results:**

A platform for remote and nondisclosive analyses of multisite individual-level data and a data catalog with information on IPEC’s variables and harmonization procedures were built. The 6-step design, harmonization procedure, ethical and legal procedures, future organizational structure and how to join IPEC were described. Data harmonization of variables of the 2 proof-of-principle cohorts was successful.

**Conclusions:**

IPEC has created a virtual databank for individual-level data of psychosis cohorts and implemented a technical infrastructure for remote federated analysis. This databank facilitates future large-scale collaborative international psychosis epidemiology research.

## Introduction

Worldwide, many research cohorts of individuals with a psychotic disorder (hereafter also referred to as psychosis) exist. Over the past decades, epidemiological research with these cohorts has greatly advanced our understanding of the distribution and mechanisms of psychotic disorders. For example, cohort studies demonstrated that the incidence of psychosis varies greatly across countries and geographical- and social contexts.^1,2^ Longitudinal studies have reported symptomatic and psychosocial recovery and remission rates in people with psychosis over time and established that, for example, negative symptoms at baseline, age of diagnosis, and duration of untreated psychosis are important markers of these outcomes.^3–5^ More recently, cohort data has been used to develop and validate models that predict outcomes in first-episode psychosis (FEP) patients, which may ultimately guide treatment planning.^6–8^

Most psychosis cohorts, however, are limited to a single setting or area, have a relatively small sample size, and cannot be compared directly because of methodological differences, such as differences in assessment instruments and follow-up period.^9–12^ Conducting studies at a larger scale is extremely costly and challenging in terms of logistic, ethical, and legal matters. Harmonizing measures from different sources can lead to an efficient and ethical use of existing data. We believe this is needed to advance the field of psychiatric epidemiology. In 2020, the Schizophrenia International Research Society (SIRS) facilitated an Epidemiology Research Harmonization Group (E-RHG), aiming to lay the foundations for prospective and retrospective data harmonization. Here, we report on the development and procedures of an initiative for retrospective data harmonization: the SIRS E-RHG-affiliated International Psychosis Epidemiology Consortium (IPEC) virtual databank.

The International Psychosis Epidemiology Consortium aims to combine existing data from psychosis cohort studies at an individual level. This approach has multiple advantages: it allows for examining (early) psychosis determinants with greater precision, including rare exposures and outcomes, comparing geographical and sociocultural contexts, and it makes optimal use of existing data. Combining individual-level data can be achieved using a virtual databank. A key difference with a more traditional nonvirtual databank is that the individual patient data does not leave the respective institutions, but instead, analyses are carried out remotely using federated analytics. In other words: analytical insight is generated from the combined information in the datasets of the cohorts via remote and nondisclosive analyses. Advantages include increased data accessibility, extensive privacy protection mechanisms, and the possibility for international researchers to work together on analyses while the individual patient data remains secure at the local institutions. Previous cross-country projects using this approach, such as the BioShaRe^13^ and the LifeCycle Project^14,15^ have been successful in developing tools for data harmonization and setting up federated platforms in population-based studies, and in pregnancy and childhood cohorts, respectively.^13–15^ A similar virtual approach is new in psychosis research.

The International Psychosis Epidemiology Consortium’s first aim is to set up the infrastructure of a virtual databank for retrospective harmonization of psychosis cohort data and harmonize data from 2 proof-of-principle psychosis cohorts. The second aim is to initiate an online platform for IPEC’s cohorts for cataloging metadata of available variables and to make harmonizable data findable and accessible to the larger research community. This profile paper describes the design and harmonization procedures, and the technical, ethical, and legal steps taken to set up this virtual databank, as well as the organizational structure we developed for IPEC. Basic descriptive data from 2 proof-of-principle cohorts in the newly developed virtual databank is presented as an illustration.

In short, this paper’s methods section provides information on the IPEC collaborative network, the broad decision-making process, the user requirements of the virtual databank, and the in- and exclusion criteria of IPEC. The results section includes the step-by-step design and harmonization procedures manual based on our experience of building the databank and details the data catalog, technical infrastructure, future organizational structure, and ethical and legal procedures.

## Methods

### The International Psychosis Epidemiology Consortium Collaborative Network

The International Psychosis Epidemiology Consortium is established to build an international, open, and collaborative psychosis research network of psychosis cohorts. The consortium is striving for global representation. The International Psychosis Epidemiology Consortium is affiliated with the SIRS E-RHG, representing cohorts from Africa,^16,17^ Asia,^16,18^ Australia,^19^ Europe,^20–24^ and South America.^16^

Within the IPEC collaborative network, a working group was formed for the proof-of-principle study with members of 2 cohorts: Psychosis Cohort Northern Netherlands (PSYCONN) and OPUS. First, within the working group, consisting of researchers from PSYCONN and OPUS, a consensus was reached about the harmonization procedures. Second, decisions were presented to the Retrospective Data Harmonization Work Package team of the SIRS E-RHG for feedback and approval.

### Requirements and In- and Exclusion Criteria

User requirements of the databank were established as follows: (1) allow for retrospective harmonization of individual-level data on psychosis, (2) enable studying of epidemiological research questions on psychosis, (3) include cohorts worldwide, (4) create a virtual databank that can be accessed by researchers worldwide, (5) comply with international ethical and legal regulations, (6) be user-friendly, and (7) be affordable to set up and maintain.

Inclusion criteria for individual cohorts were: (1) longitudinal cohort data on participants with FEP, recent-onset psychosis (< 5 years), or first presentation at a mental health institution with psychosis, (2) a diagnosis of any psychotic disorder (ICD-10 codes F10-19 subcodes with psychotic symptoms, F20-29, and F30-39 subcodes with psychotic symptoms, or ICD-9 and DSM-III/-IV/-5 equivalent [Supplement 1]), (3) ≥ 18 years of age. Inclusion criteria were relatively broad, to allow for the answering of a wide array of research questions in the future. Individual-level data of participants with a primary diagnosis of psychosis due to a physiological condition, and participants < 18 years of age was excluded. We aim to extend the inclusion criteria for future projects (eg, also include nonlongitudinal data and participants aged under 18), so that a wider variety of psychosis cohorts can join the IPEC collaborative network.

### OPUS and PSYCONN Cohorts

A virtual databank for the inclusion of pseudonymized individual patient data has been built using OPUS and PSYCONN as proof-of-principle cohorts. OPUS is a Danish Randomized Controlled Trial, investigating the effect of specialized early intervention psychosis services, for which recruitment of 18- to 45-year-old patients with first-episode schizophrenia spectrum disorder took place between 1998 and 2000 in Copenhagen and Aarhus.^20,21^ Follow-up assessments were conducted for up to 20 years.^25^ PSYCONN is a combination of 3 datasets from the northern part of the Netherlands: PROGR-S (= Psychosis Recent Onset GRoningen Survey) and PHAMOUS (= Pharmacotherapy Outcome and Monitoring Survey), both ongoing prospective cohort studies, and healthcare data from the PCR-NN (Psychiatric Case Register North-Netherlands).^22,23^ PROGR-S, initiated in 1997 without an age restriction, collects diagnostic measurements of patients with a suspected recent-onset psychotic episode or recurrent psychotic episode not diagnosed as such before.^22^ PHAMOUS, initiated in 2006, contains follow-up data of patients ≥ 18 years of age with a psychotic disorder.^23^ PCR-NN contains health care consumption, sociodemographic, and diagnosis data. PSYCONN baseline data is collected from PROGR-S and PCR-NN. PSYCONN outcome data is collected from PHAMOUS, PCR-NN, and an additional medical records file search. More detailed information on each cohort can be found in their profile papers.^20–23^

### Virtual Databank and Federated Analysis

Based on previous harmonization projects,^13–15^ literature on retrospective harmonization, including the Maelstrom Research guideline for retrospective data harmonization,^26^ and information available from companies that offer technical solutions for retrospective data harmonization (MOLGENIS^27,28^ and DataSHIELD^29,30^), a step-by-step data harmonization procedure was constructed, also as a blueprint for future work. The technical infrastructure for this virtual databank, which allows federated analytics, was developed in collaboration with the MOLGENIS and RoQua teams of the University Medical Center Groningen (UMCG). MOLGENIS offers a platform with software applications for researchers, provides expertise and support for those using the platform, and hosts the central analysis set-up of the IPEC virtual databank.^27,28^ RoQua is a routine outcome monitoring initiative supporting among others the PHAMOUS cohort.^31^ To illustrate the steps needed for retrospective data harmonization, descriptive statistics of the harmonized baseline variables “age at inclusion,” “sex,” “marital status,” and “Global Assessment of Functioning (GAF),” and of the “follow-up variable GAF” from OPUS and PSYCONN were calculated.

## Results

### Design and Harmonization Process (Figure 1)

The replicable step-by-step procedure of the design and harmonization process resulting from the collaborative process described in the methods is provided below.

**Figure 1.**
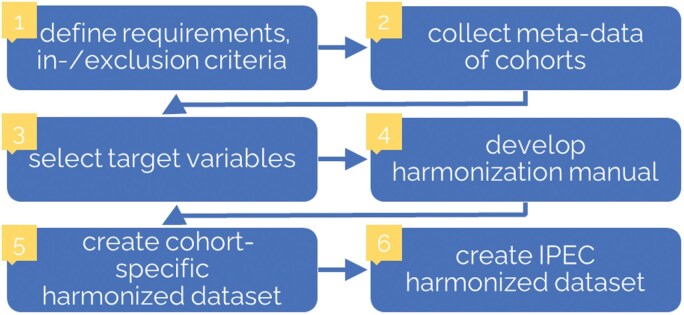
Step-by-step Design and Harmonization Process.

Step 1: Define requirements and set in-and exclusion criteria (see Methods).

Step 2: Collect metadata of cohorts

The cohorts were asked to provide detailed information on study design, population characteristics, methods, ethical procedures, and all variables in their dataset. Researchers shared an SPSS or Excel file with only metadata from their cohort, providing information on measures, variable characteristics, and value labels.

Step 3: Select target variables for harmonization

A preliminary set of target variables, also called the core dataset, was defined based on literature, expert opinion from members of the working group and the Retrospective Data Harmonization Work Package team, and metadata of the cohorts. It consisted of known and potential psychosis outcome predictors, other important baseline characteristics, outcomes related to functioning, personal well-being and symptoms, common confounders in psychosis epidemiology research, and metadata variables. The steps taken to create this core dataset were: (1) Domains were established by the working group: sociodemographic and family characteristics, social functioning, diagnostic information on the psychotic disorder and (other) clinical characteristics for baseline data, and functioning, personal outcome, and symptoms for follow-up data. (2) Target variables within these domains were discussed during working group meetings. For example, in the case of sociodemographic and family characteristics, these were: sex, gender, age, ethnicity, urbanicity, family history of mental illness, and education of parents. (3) For each target variable, the metadata of the cohorts was further detailed in the Excel inventory, including information on variable names, labels, and values (see Supplement 2). If needed, during working group meetings, researchers from both cohorts provided additional information on how their variables were constructed. (4) Based on this information, a final list of target variables was constructed, after the removal of potential target variables that were not available in both cohorts (Table 1). It was decided to harmonize data at 1, 2, 5, and 10-year follow-up. Other follow-up durations can be included when more cohorts are added to IPEC.

**Table 1.** Target Variables for Data Harmonization Selected in the Proof-of-principle Cohorts

**Baseline variables**	
Sociodemographic and family characteristics	Sex; gender
	Age at inclusion
	Part of minority group
	Highest educational degree of parent or main provider
Social functioning	Highest level of education completed
	In paid employment
	Studying
	Married
	Living situation/housing
	Global Assessment of Functioning (GAF)
Information on psychotic disorder	Age of onset current psychotic episode
	Duration of Untreated Psychosis (DUP)
	First-Episode Psychosis (FEP); number of psychotic episodes if not FEP
	Type of psychotic disorder diagnosis
	Comorbid psychiatric diagnosis
(Other) clinical characteristics	Psychotic symptoms (PANSS, SAPS/SANS)
	Comorbid diagnosis of depressive disorder
	Prior to psychosis any suicide attempt or self-harm
	Present substance abuse or dependency (alcohol, cannabis, other drugs); present cannabis use
	Use of antipsychotic medication
**Outcome variables**	
Functioning	In paid employment
	Studying
	Living situation/housing
	Social contacts: problems with relationships, satisfied with number/quality of friendships, met a friend last week, have a close friend, satisfied with family situation
	GAF
Personal outcome	Quality of life
Symptoms	Psychotic symptoms (PANSS, SAPS/SANS)
**Meta variables**	
Cohort-specific	Participant number
	Institution
	Year/month of inclusion
	Year/month of follow-up
Project-specific	Cohort ID
	Cohort country
	Study type
	Study arm

Step 4: Develop the harmonization manual

During working group meetings, consensus was sought on how the target variables across cohorts should be harmonized to make them interoperable. A harmonization manual was created, describing how to convert (also known as [a.k.a.] map) the variables from the different cohorts (a.k.a. source variables) into 1 common format (a.k.a. target format). Where possible, the definition of the target variables and the mapping were based on international standards and classification schemes (eg, the International Standard Classification of Education (ISCED) for level of education).^32^ The method used for variable mapping depended on the nature of the variables. For categorical variables such as sex or continuous variables such as the GAF, where the categories or ranges were relatively similar, simple recoding of the categories into the target format, that is, algorithmic transformation, was sufficient. Published algorithms were used when available, for example, a previously published harmonization algorithm was used for the PANSS (from PSYCONN) and the SAPS/SANS (from OPUS).^33^ Other data processing options, such as equipercentile linking, calibration models, latent variable models, and multiple imputation models were not needed for the target variables in the current dataset but might be required in future work.^26^  Supplement 3 shows an example from the harmonization manual.

Step 5: Create the cohort-specific harmonized dataset

A harmonized dataset was created by running an SPSS script separately for each cohort at the local institution, which processed the variables from the source format to the target format. Both datasets were not completely written in English and therefore had to be translated into English by the respective cohort’s researcher(s). Cohort-specific quality checks, in which data was checked for outliers, improbable values, other inconsistencies, and (number of) missing data, were done on the cleaned datasets. Datasets from cohorts joining IPEC will also be checked in the same way and if necessary, data will be corrected and changes will be documented.

Step 6: Create the IPEC harmonized dataset

Each cohort uploaded their harmonized dataset to the data portal (Armadillo server) of their respective institution’s set-up. MOLGENIS Armadillo is software for privacy-protecting statistical analyses using the DataSHIELD protocol. The datasets from the different cohorts were linked through the federated infrastructure (see Technical infrastructure). Central data quality checks were done by creating summary statistics for each variable. Again, this did not reveal data quality issues. In case of uncertainties or inconsistencies in future datasets, the corresponding research group will be contacted to provide further explanation. In case of large differences in data between cohorts, the harmonization procedure will be rediscussed and checked for potential errors, and documented.

Steps 2–6 will be repeated and the harmonization manual will be updated when deemed necessary when new cohorts and new variables are added.

#### Federated Data Analysis.

Table 2 provides the descriptive statistics calculated on the cohort-specific harmonized datasets, as well as on the combined IPEC harmonized dataset. The total IPEC sample included 1518 participants at baseline (OPUS: *N* = 578; PSYCONN: *N* = 940). The median age at inclusion was 25.6 years. Most participants were male (66.4%) and unmarried (94.4%). The mean GAF score at baseline was 44.2, and 47.0 at 1-year, 50.0 at 2-year, 52.7 at 5-year, and 51.9 at 10-year follow-up. Supplement 4 contains the R script for the descriptive analyses performed in this proof-of-principle study.

**Table 2.** Descriptive Characteristics of the Initial Variables and Cohorts Included in the IPEC Virtual Databank

Characteristics	OPUS *N* = 578	PSYCONN *N* = 940	IPEC^a^ *N* = 1518
Baseline			
Age at inclusion (years), mean (SD); median (IQR)	26.5 (6.3); 25.0 (22.0–30.0)	29.0 (9.3); 26.0 (22.0–34.0)	28.1 (8.4); 25.6 (22.0–32.5)
Female sex, *N* (%)	235 (40.7)	274 (29.2)	509 (33.6)
Married, *N* (%)	31 (5.4)	48 (5.8)	79 (5.6)
GAF, mean (SD); median (IQR)	32.1 (9.8); 31.0 (25.0–38.0)	51.9 (14.4); 50.0 (40.0–60.0)	44.2 (16.0); 42.6 (34.2–51.5)
Outcome			
GAF 1 year, mean (SD); median (IQR)	44.0 (14.1); 40.0 (35.0–55.0)	56.2 (15.1); 55.0 (46.0–65.0)	47.0 (15.3); 43.7 (37.7–57.5)
GAF 2 year, mean (SD); median (IQR)	48.0 (15.6); 45.0 (35.0–60.0)	56.1 (13.5); 55.0 (45.0–65.0)	50.0 (15.5); 47.5 (37.5–61.2)
GAF 5 year, mean (SD); median (IQR)	50.9 (17.1); 50.0 (36.3–63.0)	56.2 (14.2); 55.0 (50.0–65.0)	52.7 (16.4); 51.7 (40.9–63.7)
GAF 10 year, mean (SD); median (IQR)	50.8 (16.4); 50.0 (38.0–61.0)	55.3 (10.5); 55.0 (50.0–60.3)	51.9 (15.3); 51.2 (41.0–60.8)

Abbreviations: GAF—Global Assessment of Functioning, IQR—interquartile range, *N*—number of participants at baseline, SD—standard deviation.

^a^IPEC currently includes OPUS and PSYCONN. Therefore this column shows the combined OPUS-PSYCONN data.

Descriptives are based on valid (nonmissing) data only.

#### Data *Catalog* and *Website.*

In the open and publicly accessible data catalog, information on IPEC, and the cohorts and institutions participating in IPEC, can be found. Furthermore, detailed metadata information on the source variables, the mapping process, and a description of the target format are provided. The data catalog allows for browsing through IPEC’s current variables and selecting variables for future projects. The data catalog can be accessed via IPEC’s website: www.ipec-project.com or directly via https://data-catalogue.molgeniscloud.org/. The IPEC website also contains information on how to join IPEC, the harmonization manuals, and publications.

### Technical Infrastructure

#### Federated platform.

**Figure 2.**
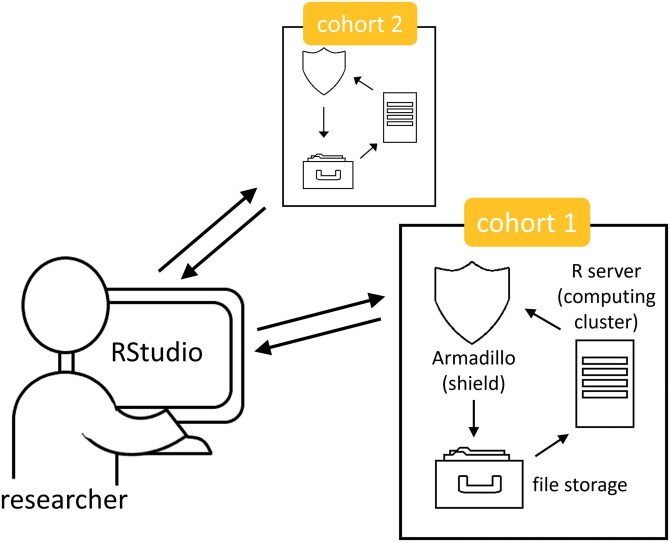
Schematic Representation of the Federated Platform. Explanation: The CAS connects the researcher with 1 or multiple Armadillo servers. A subset of the data containing the required variables is loaded into a folder on the Armadillo server specifically created for the researcher. The researcher uses RStudio and requests, for example, the mean of the variable age at inclusion. The software will then check in the file storage, within each cohort’s node (= server where the Armadillo suite lives), whether age at inclusion is available. If available, this data is transferred to the computing environment (= R servers to run the analysis on this data). Then, the mean of age at inclusion is calculated and checked for disclosive information. Next, this information goes out of the node and gives the researcher the aggregated mean of age at inclusion. Once the data is uploaded on the Armadillo server, this whole process is automated by the software and does not require local data managers to conduct analyses or aggregate data for the researcher.

A federated platform comprising the software MOLGENIS Armadillo and DataSHIELD was implemented (Figure 2). DataSHIELD is an open-source R-based tool which allows researchers to send analysis commands to the data sources and receive nondisclosive summary statistics.^29,30^ Within this platform, data remains stored on local servers of participating institutions and is not transferred between institutions. This provides data security by preventing researchers viewing the local individual-level data of other institutions.

In IPEC, DataSHIELD is implemented as follows. First, all participating institutions set up the server-side infrastructure which consists of file storage, an ID provider, and MOLGENIS’ Armadillo (a light-weight, user-friendly server that implements DataSHIELD). Second, a Central Analysis Server (CAS), which contains an RStudio environment from which researchers can perform analyses using DataSHIELD, is hosted by MOLGENIS. The CAS adds an extra layer of security, as (1) it can only be accessed by authorized researchers using authentication via FusionAuth, and (2) DataSHIELD access to the participating cohorts’ servers is only possible via the CAS. The full information flow is shown in Figure 2. A recently published article by Cadman et al. also shows the information flow in their figure 1.^34^ Furthermore, another recent paper by Avraam et al. explains DataSHIELD’s disclosure prevention mechanisms.^35^

Requests for access to the virtual databank can be submitted to the steering committee. The central data manager will keep a log of which researchers are granted access by the steering committee and discuss this with the local data managers. Next, the local data managers create different projects with subsets of the data and assign permissions for specific projects to researchers on the local Armadillo. For each research project conducted on the data in the virtual databank, the local data manager of each cohort uploads a cleaned dataset in harmonized format via R to the local Armadillo server. To analyze data, researchers have to log in on the CAS that federates to all of the institutions via LifeScience AAI service with the account belonging to their employer/institute. In the CAS, researchers run analysis scripts in RStudio on the portion of local data assigned to them. The analysis requests are sent from the CAS to the participating local instances, datasets are analyzed simultaneously, and the results of the analyses on each cohort’s dataset are combined and returned to the researcher as aggregate data. The federated platform allows for both running separate analyses on each local instance, as well as coanalyzing data from multiple sources together. Furthermore, a range of analyses, such as descriptives, generalized linear models, mediation analyses, and survival models can be performed. For a full list of available functions within the DataSHIELD client packages, please view the DataSHIELD documentation via: https://cran.datashield.org/web/.

### IPEC Organizational Structure

**Figure 3.**
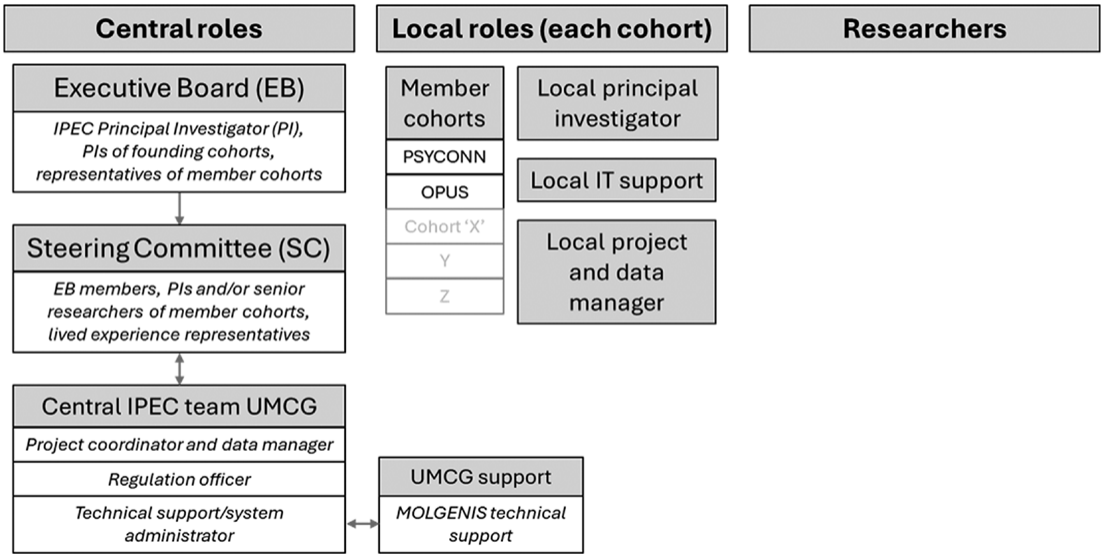
Organizational Roles in the IPEC Federated Network.

Based on our learnings from creating the IPEC virtual databank, we have developed a high-level organizational structure for IPEC at central (consortium) and local (cohort) levels for future work. This structure is displayed in Figure 3.

Three central organizational structures within IPEC ensure coordinated controlled workflow: the executive board, the steering committee, and the central IPEC team UMCG. The executive board consists of the IPEC Principal Investigator (PI) (chair), PIs of the founding cohorts (PSYCONN, OPUS, INTREPID^16^), and selected representatives from member cohorts. The executive board is responsible for strategic decision-making, budget and funding decisions, and approval of project requests and publication proposals. Membership is on invitation, for the duration of 5 years (renewable term) or until the end of cohort membership. The executive board should meet at least twice per year. The steering committee consists of executive board members, PIs and/or senior researchers (early career or midcareer) from member cohorts, and representatives of people with lived experience of psychosis. The steering committee, among other things, advises on new and ongoing projects, and decides on selecting target variables, data pooling, and harmonization. Membership is on invitation. Member cohorts are requested to propose candidates. The steering committee should meet at least 4 times per year. The Central IPEC team UMCG consists of the IPEC project coordinator and data manager, the IPEC regulation officer, and the IPEC technical support/system administrator.

Additional UMCG support will be available; MOLGENIS will provide second-line technical support in collaboration with the IPEC technical support/system administrator. Second-line support may involve solving complex problems during the installation of the Armadillo and DataSHIELD software and maintenance of the central authentication mechanism and central analysis server, as well as monitoring operating systems used to run the federated platform.

The local organizational structure consists of a local principal investigator, local IT support, and a local project and data manager.

#### New *Member Cohorts.*

Eligible psychosis cohorts can request to join IPEC. The central IPEC data manager will guide them through the application process. After submitting a completed checklist, the steering committee will decide on membership. Membership will start after the consortium agreement is signed. Member cohorts should have a local PI, a local project and data manager, and local IT support staff.

### Access to the IPEC Virtual Databank by Researchers

The International Psychosis Epidemiology Consortium’s harmonized cohort data is available for research projects by researchers who are and who are not affiliated with member cohorts. Researchers interested in using data from the virtual databank can access the IPEC data catalog and file a project request in which they, among others, include which cohorts and variables they are interested in. A template project request can be obtained via the IPEC website. After checking whether the proposed research question falls within the scope of IPEC and whether a similar request has not previously been approved, the central data manager forward the request to the PI and data manager of the cohorts involved. If all parties are interested and willing to participate, the project request is submitted to the executive board for final approval. After approval, for all new projects, a data access agreement will be signed with all parties involved. The central IPEC regulation officer will provide the templates for agreements. Finally, local data managers will grant the researcher user rights to a project-specific dataset in their local Armadillo environment (again, the researcher will not be able to view or access the individual patient data), and the researcher will be given access to the analysis environment of the virtual databank.

### Ethical and Legal Procedures

The UMCG board of directors granted permission to set up this virtual databank. The research plan was approved by the UMCG Central Ethics Review Board and registered in the UMCG Research Register (RR 201900598, PaNaMa 8556). A Privacy Impact Analysis (PIA) was conducted in the UMCG and approved by the privacy officer. For OPUS, the Danish data protection agency granted permission to share data for this project and the National Archives transferred the data to OPUS. The technical set-up was approved by the IT department in the Capital Region. A research agreement was set up between the participating institutions. To protect the participants’ privacy, extensive safeguards were put in place (see section “Technical infrastructure”).

Each cohort participating in IPEC is responsible for checking their institution’s and country’s ethical and legal regulations and making sure these are met, as well as sharing this evidence with IPEC’s central regulation officer. These responsibilities include checking whether a PIA or a similar procedure is required to provide insight into any risk concerning the data protection for the persons involved and the organization, the scope of the risk, and any required measures. Cohorts joining IPEC are also responsible for submitting a data request to their local study committee for the use of their data in IPEC if such procedure is required. If the cohort does not have the technical capacity to install the required software and ensure integration into the virtual databank, data transfer is possible. In such a case, the cohort must ensure compliance with data transfer regulations and sign a data transfer agreement with the UMCG and MOLGENIS. Furthermore, data uploaded to the Armadillo server has to be pseudonymized or anonymized. For further details on IPEC’s ethical and legal procedures see Supplement 5.

## Discussion

IPEC has cataloged and retrospectively harmonized data from 2 proof-of-principle psychosis cohorts, laying the foundation for future international collaborative research on psychosis epidemiology. The IPEC proof-of-principle study has shown that it is possible to set up a virtual databank and to implement a technical infrastructure for remote federated analysis of individual-level data of psychosis patients. Also, information on IPEC’s source and target variables, the harmonization process, and the cohorts participating in IPEC have been uploaded to an open online data catalog. The virtual databank and the data catalog contribute to the FAIR (Findable, Accessible, Interoperable, and Reusable) principles.^36^ The data catalog makes IPEC’s (meta)data findable and promotes the reusability of the data. With the federated platform, data will be more accessible to researchers due to not having to transfer data physically to 1 institution. Harmonization of variables across cohorts and countries will lead to increased interoperability.

### Methodological Discussion

Whilst consensus on the harmonization process was reached by the researchers represented in the working group, this was done in close collaboration with each institution’s multidisciplinary cohort team. Researchers who developed or previously worked with the datasets provided detailed information on how and why certain variables were originally constructed. Involving people with different areas of expertise increased the understanding and quality of the harmonization procedure. Also, this project builds on previously conducted harmonization projects in other research fields and harmonization guidelines.^13–15,26^ There is substantial variation in variables between studies, for example, categorical versus continuous, categorical but with different categories, self-reported versus clinician-reported, and different variable definitions. This heterogeneity was discussed during the IPEC working group meetings. For example, for the harmonized variable “married,” OPUS and PSYCONN had different source data available (OPUS: unmarried, married, separated/divorced, widowed; PSYCONN: married, living alone, living with parents, living with partner or family, mental health institute, homeless). Therefore, only a distinction between married and all other categories could be made. Although this leads to information loss, the harmonized variable was still considered relevant to the IPEC research question and was therefore included. Currently, a limited number of variables and cohorts have been included in the virtual databank. This allowed us to ensure the federated platform runs smoothly, before adding more variables and cohorts. The proof-of-principle cohorts OPUS and PSYCONN have both been previously used in research, include a relatively large number of participants at baseline, and have many baseline and outcome variables available. These cohorts, therefore, provided a good base for the IPEC virtual databank. Most of the software used for the federated network is open-source. Nevertheless, experts on (the IT part of) federated data analysis were consulted to assist in setting up the technical infrastructure and designing it specifically around IPEC’s requirements. It is important that every cohort involves their IT department and consults their ethical and legal departments about which regulations apply. Cohorts that want to join IPEC in the future will enter the Joint Consortium Agreement. IPEC will guide its partners through the steps required to set up and maintain the federated network.

### Future Perspectives and Recommendations

The IPEC virtual databank will allow combining large-scale individual patient data and international collaborative research on (early) psychosis. In the coming years, more variables and cohorts will be added to the databank, increasing the representation of (early) psychosis cohorts in the world. The harmonization process will be further streamlined to ensure feasible implementation and sustainable growth of IPEC. Ultimately, the aim is to create a data-sharing platform for psychosis cohorts from different settings all over the world. Special attention will be paid to including cohorts from low- and middle-income countries (LMICs). We will draw on ethics lessons shared by the Africa Ethics Working Group (AEWG), such as involving experts in ethics from the local or country level of a participating cohort, and recognizing the vulnerability to inequity in research partnerships with countries from LMICs and addressing this accordingly.^37^

## Supplementary Material

Supplementary material is available at https://academic.oup.com/schizophreniabulletin.

sbaf094_suppl_Supplementary_Material
