# Supplementary material for: The International Psychosis Epidemiology Consortium Virtual Databank—A Platform for Data Harmonization and Federated Analysis of Psychosis Cohorts
Source: Schizophr Bull. 2025 Jul 17;52(4):sbaf094. doi: 10.1093/schbul/sbaf094 (PMC13391649; doi:10.1093/schbul/sbaf094)
Supplement: sbaf094_suppl_Supplementary_Material [file sbaf094_suppl_supplementary_material.docx]

**Supplementary materials**

***The International Psychosis Epidemiology Consortium virtual databank – a platform for data harmonization and federated analysis of psychosis cohorts***

Supplement 1. Diagnoses included in IPEC.

Supplement 2. Additional information on cohorts' source variables in the inventory.

Supplement 3. Example from the harmonization manual: Global Assessment of Functioning (GAF).

Supplement 4. R script for the descriptive analyses.

Supplement 5. Ethical and legal procedures – additional information.

Supplement 6. Collaborators in the SIRS Epidemiology Research Harmonization Group.

Supplement 1. Diagnoses included in IPEC.

IPEC includes individuals with the following diagnoses: ICD-9 codes 291.3, 291.5, 292.1, 293.81, 293.82, 295, 296 with psychotic behavior, 297, 298, and 301.22, ICD-10 codes F10-19 subcodes with psychotic symptoms, F20-29, and F30-39 subcodes with psychotic symptoms, and DSM-III/-IV/-5 criteria for any diagnosis of schizophrenia spectrum or other psychotic disorder (incl. substance/medication-induced), bipolar disorder or major depressive disorder with psychotic features, schizotypal personality disorder, and catatonia.

Supplement 2. Additional information on cohorts' source variables in the inventory.

| Name cohort | Variable name | Variable label | Values and value labels | Unit | Value type | Measure | Missing code | Informant | Comments/ further instructions |
| --- | --- | --- | --- | --- | --- | --- | --- | --- | --- |
|  | The variable name in the dataset | Description of variable | The categories for categorical variables | The units for continuous variables (e.g. years, months, days, kg, g, m, cm) | Integer or Decimal:  - Integer: accepts positive and negative whole numbers, but not decimals or fractions  - Decimal: accepts any number with decimal places | The data type: scale (interval or ratio), ordinal, nominal, string | Values for missing | E.g. clinician, self-reported, family member |  |

Supplement 3. Example from the harmonization manual: Global Assessment of Functioning (GAF).

*Domain:*

GAF

*Source information:*

| Name cohort | Variable name | Variable label | Values and value labels | Unit | Value type | Measure | Missing code | Informant | Comments/  further instructions |
| --- | --- | --- | --- | --- | --- | --- | --- | --- | --- |
|  | The variable name in the dataset | Description of variable | The categories for categorical variables | The units for continuous variables (e.g. years, months, days, kg, g, m, cm) | Integer or Decimal:  - Integer: accepts positive and negative whole numbers, but not decimals or fractions  - Decimal: accepts any number with decimal places | The data type: scale (interval or ratio), ordinal, nominal, string | Value for missing | E.g. clinician, self-reported, family member |  |
| PROGRs (PSYCONN) | GAF | Global Assessment of Functioning | NA | NA | integer | scale | NS | NS | NS |
|  | DSMIV_5 | DSM-IV Diagnosis Axis 5 GAF score (0-100) | NA | NA | integer | scale | 999 | research nurse | 0=inadequate information |
| PHAMOUS (PSYCONN) | gaf_symp | GAF Symptoms | NA | NA | integer | scale | 999 | NS | NS |
|  | gaf_dis | GAF Disability | NA | NA | integer | scale | 999 | NS | NS |
| OPUS | gaf1.1 | Baseline Aktuelt: Symptoms | NA | NA | integer | scale | NS | research rating | NS |
|  | gaf1.2 | Baseline Aktuelt: Funktion | NA | NA | integer | scale | NS | research rating | NS |

NA – not applicable; NS – not specified

*Target variable:*

| Variable name | Variable label | Values and value labels | Unit | Value type | Measure | Missing code | Informant | Comments/  further instructions |
| --- | --- | --- | --- | --- | --- | --- | --- | --- |
| The variable name in the dataset | Description of variable | The categories for categorical variables | The units for continuous variables (e.g. years, months, days, kg, g, m, cm) | Integer or Decimal:  - Integer: accepts positive and negative whole numbers, but not decimals or fractions  - Decimal: accepts any number with decimal places | The data type: scale (interval or ratio), ordinal, nominal, string | Value for missing | Informant  E.g. clinician, self-reported, family member |  |
| gafbaseline | Global Assessment of Functioning at baseline | -999=Missing | NA | integer | scale | -999 | NS | NS |

NA – not applicable; NS – not specified

*Harmonization description:*

- PROGRs:
  - IF SYSMIS(DSMIV_5) DSMIV_5=GAF.
  - IF (DSMIV_5=999 AND NOT(SYSMIS(GAF))) DSMIV_5=GAF.
  - gaf_sympdis=MIN(gaf_symp, gaf_dis).
  - IF ((SYSMIS(DSMIV_5) AND SYSMIS(GAF)) AND NOT (SYSMIS(gaf_sympdis))) DSMIV_5=gaf_sympdis.
  - RECODE DSMIV_5 (0 999=-999) (else=copy) INTO gafbaseline.
  - gafbaseline (999=-999).
- OPUS:
  - GAF_min=MIN(gaf1.1, gaf1.2).
  - gafbaseline=GAF_MIN.
  - gafbaseline=-999 if GAF_MIN=0 or missing.

*Comments:*

- PROGRs (PSYCONN): 2 variables can be used for the GAF: “GAF” and “DSMIV_5”. “GAF” is based on the GAF score from the registration of the “Diagnose Behandeling Combinatie” (in English: Diagnosis Treatment Combination; the basis of Dutch hospital care finance). “DSMIV_5” is scored by research nurses specifically for PROGRs. “DSMIV_5”, therefore, seems more reliable. “GAF” was used in case the “DSMIV_5” variable was missing. If both the “GAF” variable and the “DSMIV-5” variable were missing, the GAF score from the PHAMOUS dataset was used (only if the GAF score from PHAMOUS was obtained within 3 months from the inclusion date of the participant in PROGRs). PHAMOUS has 2 GAF variables: “GAF Symptoms” and “GAF Disability”. Following Vatnaland *et al*., the lowest score of these variables has been used to replace the missing “DSMIV_5” variable.
- OPUS has 2 GAF variables: “Baseline Aktuelt: Symptoms” (i.e.; GAF symptoms) and “Baseline Aktuelt: Funktion” (i.e.; GAF function). Following Vatnaland *et al*., the lowest score of these variables has been used to harmonize with the other cohort(s). In case one of the 2 GAF variables was missing, the score was based on the GAF variable that was present.

*Vatnaland T, Vatnaland J, Friis S, Opjordsmoen S. Are GAF scores reliable in routine clinical use? Acta Psychiatr Scand. 2007;115(4):326-330. doi:10.1111/j.1600-0447.2006.00925.x*

Supplement 4. R script for the descriptive analyses.

###installation.

#install and load required packages.

install.packages("DSI")

install.packages("DSMolgenisArmadillo")

install.packages("dsBaseClient", repos = c("http://cran.datashield.org", "https://cloud.r-project.org/"), dependencies = TRUE)

library(DSI)

library(DSMolgenisArmadillo)

library(dsBaseClient)

###get a token from the ID server.

#OPUS: specify server url.

armadillo_opus_url <- "*url can be requested*"

#OPUS: get token from central authentication server.

tokenopus <- armadillo.get_token(armadillo_opus_url)

#PSYCONN: specify server url.

armadillo_psyconn_url <- " *url can be requested* "

#PSYCONN: get token from central authentication server.

tokenpsyconn <- armadillo.get_token(armadillo_psyconn_url)

###build the login frame.

#build the login dataframe.

builder <- DSI::newDSLoginBuilder()

builder$append(server = "armadillo_opus",

url = armadillo_opus_url,

token = tokenopus,

driver = "ArmadilloDriver")

builder$append(server = "armadillo_psyconn",

url = armadillo_psyconn_url,

token = tokenpsyconn,

driver = "ArmadilloDriver")

#create loginframe.

logindata <- builder$build()

###login.

conns <- datashield.login(logins = logindata, symbol = "D")

#check available tables.

datashield.tables(conns)

#assign opus table data to a symbol.

datashield.assign.table(

conns = conns$armadillo_opus,

table = "opuswideparquit/opuswideparquetfolder/230328_opus",

symbol = "D"

)

#assign psyconn table data to a symbol.

datashield.assign.table(

conns = conns$armadillo_psyconn,

table = "psyconnparquet/psyconnparquetfolder/230328_PSYCONN",

symbol = "D"

)

#check type of R object.

ds.class(x="D")

#check column names

ds.colnames("D", datasources=conns[1])

ds.colnames("D", datasources=conns[2])

#check if D object exists on the server-side.

ds.testObjExists(test.obj.name = "D", datasources = conns)

#check whether tables exist and assignable.

datashield.table_status(conns, logindata)

###replace missing values

#replace -999 with NA in opus data

ds.recodeValues(var.name = 'D$ageinclusion', values2replace.vector = '-999', new.values.vector = 'NA', newobj='ageinclc', datasources=conns[1])

ds.recodeValues(var.name = 'D$gafbaseline', values2replace.vector = '-999', new.values.vector = 'NA', newobj='gafbasc', datasources=conns[1])

ds.recodeValues(var.name = 'D$sex', values2replace.vector = '-999', new.values.vector = 'NA', newobj='sexc', datasources=conns[1])

ds.recodeValues(var.name = 'D$married', values2replace.vector = '-999', new.values.vector = 'NA', newobj='marriedc', datasources=conns[1])

ds.recodeValues(var.name = 'D$gaf1year', values2replace.vector = '-999', new.values.vector = 'NA', newobj='gaffu1', datasources=conns[1])

ds.recodeValues(var.name = 'D$gaf2year', values2replace.vector = '-999', new.values.vector = 'NA', newobj='gaffu2', datasources=conns[1])

ds.recodeValues(var.name = 'D$gaf5year', values2replace.vector = '-999', new.values.vector = 'NA', newobj='gaffu5', datasources=conns[1])

ds.recodeValues(var.name = 'D$gaf10year', values2replace.vector = '-999', new.values.vector = 'NA', newobj='gaffu10', datasources=conns[1])

#replace -888 with NA in psyconn data.

ds.recodeValues(var.name = 'D$gaf1year', values2replace.vector = '-888', new.values.vector = 'NA', newobj='gaf1yearc', datasources=conns[2])

ds.recodeValues(var.name = 'D$gaf2year', values2replace.vector = '-888', new.values.vector = 'NA', newobj='gaf2yearc', datasources=conns[2])

ds.recodeValues(var.name = 'D$gaf5year', values2replace.vector = '-888', new.values.vector = 'NA', newobj='gaf5yearc', datasources=conns[2])

ds.recodeValues(var.name = 'D$gaf10year', values2replace.vector = '-888', new.values.vector = 'NA', newobj='gaf10yearc', datasources=conns[2])

#replace -999 with NA in psyconn data.

ds.recodeValues(var.name = 'D$ageinclusion', values2replace.vector = '-999', new.values.vector = 'NA', newobj='ageinclc', datasources=conns[2])

ds.recodeValues(var.name = 'D$gafbaseline', values2replace.vector = '-999', new.values.vector = 'NA', newobj='gafbasc', datasources=conns[2])

ds.recodeValues(var.name = 'D$sex', values2replace.vector = '-999', new.values.vector = 'NA', newobj='sexc', datasources=conns[2])

ds.recodeValues(var.name = 'D$married', values2replace.vector = '-999', new.values.vector = 'NA', newobj='marriedc', datasources=conns[2])

ds.recodeValues(var.name = 'gaf1yearc', values2replace.vector = '-999', new.values.vector = 'NA', newobj='gaffu1', datasources=conns[2])

ds.recodeValues(var.name = 'gaf2yearc', values2replace.vector = '-999', new.values.vector = 'NA', newobj='gaffu2', datasources=conns[2])

ds.recodeValues(var.name = 'gaf5yearc', values2replace.vector = '-999', new.values.vector = 'NA', newobj='gaffu5', datasources=conns[2])

ds.recodeValues(var.name = 'gaf10yearc', values2replace.vector = '-999', new.values.vector = 'NA', newobj='gaffu10', datasources=conns[2])

###ANALYSES.

###perform descriptive analysis.

#N(%).

ds.table("sexc", datasources=conns[2])

ds.table("sexc", datasources=conns[1])

ds.table("marriedc", datasources=conns[2])

ds.table("marriedc", datasources=conns[1])

ds.table("sexc", datasources=conns)

ds.table("marriedc", datasources=conns)

#calculate the mean.

ds.mean("ageinclc", type = "both", datasources = conns)

ds.mean("gafbasc", type = "both", datasources = conns)

ds.mean("gaffu1", type = "both", datasources = conns)

ds.mean("gaffu2", type = "both", datasources = conns)

ds.mean("gaffu5", type = "both", datasources = conns)

ds.mean("gaffu10", type = "both", datasources = conns)

#calculate the median and IQR.

ds.summary("ageinclc", datasources = conns)

ds.summary("gafbasc", datasources = conns)

ds.summary("gaffu1", datasources = conns)

ds.summary("gaffu2", datasources = conns)

ds.summary("gaffu5", datasources = conns)

ds.summary("gaffu10", datasources = conns)

ds.quantileMean(x = "ageinclc", type = "combine", datasources = conns)

ds.quantileMean(x = "ageinclc", type = "split", datasources = conns)

ds.quantileMean(x = "gafbasc", type = "combine", datasources = conns)

ds.quantileMean(x = "gafbasc", type = "split", datasources = conns)

ds.quantileMean(x = "gaffu1", type = "combine", datasources = conns)

ds.quantileMean(x = "gaffu1", type = "split", datasources = conns)

ds.quantileMean(x = "gaffu2", type = "combine", datasources = conns)

ds.quantileMean(x = "gaffu2", type = "split", datasources = conns)

ds.quantileMean(x = "gaffu5", type = "combine", datasources = conns)

ds.quantileMean(x = "gaffu5", type = "split", datasources = conns)

ds.quantileMean(x = "gaffu10", type = "combine", datasources = conns)

ds.quantileMean(x = "gaffu10", type = "split", datasources = conns)

#calculate the sd via variance.

varage <- ds.var("ageinclc", type = "both", checks = FALSE, datasources = conns)

varage

summary(varage)

sqrt(varage$Variance.by.Study)

sqrt(varage$Global.Variance)

vargafb <- ds.var("gafbasc", type = "both", checks = FALSE, datasources = conns)

vargafb

summary(vargafb)

sqrt(vargafb$Variance.by.Study)

sqrt(vargafb$Global.Variance)

vargaffu1 <- ds.var("gaffu1", type = "both", checks = FALSE, datasources = conns)

vargaffu1

summary(vargaffu1)

sqrt(vargaffu1$Variance.by.Study)

sqrt(vargaffu1$Global.Variance)

vargaffu2 <- ds.var("gaffu2", type = "both", checks = FALSE, datasources = conns)

vargaffu2

summary(vargaffu2)

sqrt(vargaffu2$Variance.by.Study)

sqrt(vargaffu2$Global.Variance)

vargaffu5 <- ds.var("gaffu5", type = "both", checks = FALSE, datasources = conns)

vargaffu5

summary(vargaffu5)

sqrt(vargaffu5$Variance.by.Study)

sqrt(vargaffu5$Global.Variance)

vargaffu10 <- ds.var("gaffu10", type = "both", checks = FALSE, datasources = conns)

vargaffu10

summary(vargaffu10)

sqrt(vargaffu10$Variance.by.Study)

sqrt(vargaffu10$Global.Variance)

Supplement 5. Ethical and legal procedures – additional information.

Each participating cohort is responsible for checking whether participants provided informed consent for future scientific research, including participation in a (virtual) databank, checking the objection registry, if present, and determining whether additional ethical review is warranted. Written informed consent for inclusion in the virtual databank has not been asked from the participants of the OPUS and PSYCONN cohorts, due to the disproportionate effort needed to obtain this consent (Section 7:458 (WGBO)). The nature and purpose of this research project ask for the inclusion of these datasets since the research serves a public interest and the research cannot be conducted without the relevant data. Extensive safeguards have been put in place to protect the privacy of the participants.

For the PSYCONN data, data access agreements were put in place between the institutions that provide the data (UMCG/UCP, Lentis Psychiatric Institute, GGZ Drenthe, GGZ Friesland) and the institution that manages the virtual databank (UMCG/UCP).

Supplement 6. Collaborators in the SIRS Epidemiology Research Harmonization Group.

*SIRS Epidemiology Research Harmonization Group*

| Continent | Author name | Affiliation |
| --- | --- | --- |
| Africa | Bonginkosi Chiliza | Department of Psychiatry, University of KwaZulu-Natal, Nelson R Mandela School of Medicine, Durban, South Africa |
| Africa | Oye Gureje | Department of Psychiatry, University College Hospital, Ibadan, Nigeria |
| Africa | Eleni Misganaw | Mental Health Service Users Association, Ethiopia  Global Mental Health Peer Network, Ethiopia |
| Africa | Charlene Sunkel | Global Mental Health Peer Network, South Africa |
| Asia | Eric Chen | Department of Psychiatry, LKS Faculty of Medicine, University of Hong Kong, Hong Kong  State Key Laboratory of Brain and Cognitive Sciences, University of Hong Kong, Hong Kong |
| Asia | Thara Rangaswamy | Schizophrenia Research Foundation, Chennai, Tamil Nadu, India |
| Asia/Europe | Sara Farhang | Research Center of Psychiatry and Behavioral Sciences, Tabriz University of Medical Sciences, Tabriz, Iran  University of Groningen, University Medical Center Groningen, University Center Psychiatry, Rob Giel Research Center, The Netherlands |
| Australia/Europe | Brian O’Donoghue | University College Dublin, Dublin, Ireland  Orygen, The National Centre of Excellence in Youth Mental Health, Parkville, Melbourne, Australia |
| Europe | Nikolai Albert | Copenhagen Research Center for Mental Health – CORE, Mental Health Center Copenhagen, Copenhagen University Hospital, Gentofte Hospitalsvej 15, 4, DK-2900 Hellerup, Denmark & Mental Health Centre Amager, University Hospital Copenhagen, Digevej 110, 2300 Copenhagen S, Denmark |
| Europe | Celso Arango | Department of Child and Adolescent Psychiatry, Institute of Psychiatry  and Mental Health, Hospital General Universitario Gregorio Marañón, IiSGM, CIBERSAM, School of Medicine, Universidad Complutense de Madrid, Madrid, Spain |
| Europe | Vera Brink | University of Groningen, University Medical Center Groningen, University Center Psychiatry, Groningen, The Netherlands |
| Europe | Peter B. Jones | Department of Psychiatry, University of Cambridge, Cambridge, United Kingdom  Cambridgeshire and Peterborough NHS Foundation Trust, Cambridge, England, United Kingdom |
| Europe | Hannah Jongsma | University of Groningen, University Medical Center Groningen, University Center Psychiatry, Groningen, The Netherlands  Veldzicht Centre for Transcultural Psychiatry, Balkbrug, The Netherlands |
| Europe | Ian Kelleher | Department of Child and Adolescent Psychiatry, University of Edinburgh, Edinburgh, Scotland, United Kingdom |
| Europe | James Kirkbride | University College London, Division of Psychiatry, PsyLife group, London, England, United Kingdom |
| Europe | Craig Morgan | ESRC Centre for Society and Mental Health, King’s College London; Department of Health Service and Population Research, Institute of Psychiatry, Psychology, and Neuroscience, King’s College London, London, United Kingdom |
| Europe | Robin Murray | National Institute for Health Research, Mental Health Biomedical Research Centre at South London and Maudsley NHS Foundation Trust and King’s College, London, United Kingdom  Department of Psychosis Studies, Institute of Psychiatry, Psychology and Neuroscience, King’s College London, London, United Kingdom |
| Europe | Tessa Roberts | Centre for Society & Mental Health, King's College London, United Kingdom |
| Europe | Wim Veling | University of Groningen, University Medical Center Groningen, University Center Psychiatry, Groningen, The Netherlands |
| Europe | Els van der Ven | Department of Clinical, Neuro- and Developmental Psychology, Vrije Universiteit Amsterdam, Amsterdam, The Netherlands |
| Europe | Diego Quattrone | Department of Social, Genetic and Developmental Psychiatry Centre, Institute of Psychiatry, Psychology and Neuroscience,  King’s College London, London, United Kingdom  National Institute for Health Research, Mental Health Biomedical Research Centre at South London and Maudsley NHS Foundation Trust and King’s College, London, United Kingdom  South London and Maudsley Mental Health NHS Trust, London, United Kingdom |
| North America | Kelly Anderson | Department of Epidemiology and Biostatistics, Schulich School of Medicine and Dentistry, Western University, London, Ontario, Canada |
| North America | Nev Jones | University of Pittsburgh, The School of Social Work, Pittsburgh, Pennsylvania, United States |
| North America | Ezra Susser | Department of Psychiatry, Columbia University and the New York State Psychiatric Institute, New York, New York, United States  Department of Epidemiology, Mailman School of Public Health, Columbia University, New York, New York, United States |
| South America | Cristina Marta Del-Ben | Neuroscience and Behavior Department, Ribeirão Preto Medical School, University of São Paulo, Ribeirão Preto, Brazil |
| South America | Alfonso Gonzalez-Valderrama | Early Intervention Program, Instituto Psiquiátrico Dr J. Horwitz Barak, Santiago, Chile |
| South America | Paulo Rossi Menezes | Population Mental Health Research Centre, Universidade de São Paulo, São Paulo, Brazil  Department of Preventive Medicine, Faculdade de Medicina, Universidade de São Paulo, São Paulo, Brazil |

*PROGRs Investigators*

| Author name | Affiliation |
| --- | --- |
| Edith Liemburg | Department of Research, GGZ Friesland, Leeuwarden, The Netherlands |
| Stynke Castelein | Lentis Psychiatric Institute, Lentis Research, Groningen, The Netherlands  University of Groningen, Faculty of Behavioral and Social Sciences, Department of Clinical Psychology and Experimental Psychopathology, Groningen, The Netherlands  University of Groningen, University Medical Center Groningen, Rob Giel Research Center, Groningen, The Netherlands |
| Wim Veling | University of Groningen, University Medical Center Groningen, University Center Psychiatry, Groningen, The Netherlands |

*PHAMOUS Investigators*

| Author name | Affiliation |
| --- | --- |
| Edith Liemburg | Department of Research, GGZ Friesland, Leeuwarden, The Netherlands |
| Ellen Visser | University of Groningen, University Medical Center Groningen, University Center Psychiatry & Rob Giel Research Center, Groningen, The Netherlands |
| Frederike Jörg | University of Groningen, University Medical Center Groningen, Interdisciplinary Center Psychopathology and Emotion Regulation (ICPE) & Rob Giel Research Center, Groningen, The Netherlands |
| Gerdina Hendrika Marieke Pijnenborg | University of Groningen, Faculty of Behavioral and Social Sciences, Department of Clinical Psychology and Experimental Psychopathology, Groningen, The Netherlands |
| Wim Veling | University of Groningen, University Medical Center Groningen, University Center Psychiatry, Groningen, The Netherlands |
